# Novel seed generation and quadrature-based square rooting algorithms

**DOI:** 10.1038/s41598-022-25039-y

**Published:** 2022-11-29

**Authors:** Amal Altamimi, Belgacem Ben Youssef

**Affiliations:** 1 Department of Computer Engineering, College of Computer & Information Sciences, King Saud University, P.O. Box 51178, Riyadh, 11543 Saudi Arabia; 2 National Satellite Technology Center, Space and Aeronautic Research Institute, King Abdulaziz City for Science and Technology, P.O. Box 8612, Riyadh, 12354 Saudi Arabia

**Keywords:** Engineering, Mathematics and computing

## Abstract

The square root operation is indispensable in a myriad of computational science and engineering applications. Various computational techniques have been devised to approximate its value. In particular, convergence methods employed in this regard are highly affected by the initial approximation of the seed value. Research shows that the provision of an initial approximation with higher accuracy yields fewer additional iterations to calculate the square root. In this article, we propose two novel algorithms. The first one presents a seed generation technique that depends on bit manipulation and whose output is to be used as an initial value in the calculation of square roots. The second one describes a quadrature-based square rooting method that utilizes a rectangle as the plane figure for squaring. We provide error estimation of the former using the vertical parabola equation and employ a suitable lookup table, for the latter, to store needed cosine values. The seed generation approach produces a significant reduction in the number of iterations of up to 84.42% for selected convergence methods. The main advantages of our proposed square rooting algorithm lie in its high accuracy and in its requirement of just a single iteration. Our proposed algorithm also provides for lower computational latency, measured in the number of clock cycles, compared to Newton–Raphson’s and Bakhshali’s square rooting methods.

## Introduction

Computing the square root is highly useful in a variety of science and engineering areas. In particular, it is an essential operation in Digital Signal Processing (DSP) and many applications in control systems^1,2^. Indeed, the IEEE 754 standard, revised in 2019, classifies the square root operation as one of the five basic arithmetic operations besides addition, subtraction, multiplication, and division^3^. Despite the fact that a hardware-implemented square root operation has a lot in common with division, it remains one of the most expensive operations due to the complexity of its algorithms^4^.

Generally, square rooting methods can be classified into subtractive, multiplicative, and approximation methods. In addition, bit-manipulation techniques are used to provide a rough approximation of the square root value. These techniques utilize properties of the binary representation to perform tasks such as counting the leading or trailing zeros, extracting contiguous bits, and locating the first or last set bit, among others^5,6^. Further elaboration on the use of bit-manipulation techniques to perform different arithmetic operations digitally is provided in^7–9^.

Subtractive methods (a.k.a digit-recurrence or digit-by-digit methods) compute the square root directly one digit at a time, starting from the most significant digit. The main advantages of such methods are the absence of division operation and the generation of accurate results^1^. On the other hand, the downside of this type of algorithm is its slow convergence resulting in increased computational time^1^. The pencil-and-paper method is a well-known technique under this category^10^. Typically, digit-recurrence methods are used with hardware devices such as microcontrollers as well as the early generations of Field-Programmable Gate Arrays (FPGAs), where no dedicated multipliers were included^11^. Two main variations of digit-recurrence algorithms are restoring and nonrestoring algorithms. In the restoring method, as the name implies, the partial remainder is restored to its value in the previous iteration when it becomes negative. Consequently, the worst case of the restoring method requires two arithmetic operations per quotient digit^12^. This method needs extra hardware resources and tends to lengthen the clock cycle compared to the nonrestoring method^10,13^. On the other hand, the latter method temporarily allows for an incorrect partial remainder and does not require the restoring step, hence the name^10^. The incorrect partial remainder is then corrected in the next cycle by adding the divisor instead of subtracting it from the partial remainder^14^. The nonrestoring method can further be divided into techniques with non-redundant and redundant digit sets^15^. The use of the redundant digit sets has the advantage of avoiding the long carry-propagated additions introduced using those sets of digits.

Unlike subtractive methods, multiplicative methods (a.k.a iterative or convergence methods), such as Newton–Raphson, Goldschmidt, and Bakhshali’s algorithms, are characterized by faster convergence^16^. Besides, digits are not computed directly, but an initial estimation is refined progressively until the desired accuracy is reached^2^. Further, the choice of this initial value determines the speed of calculations^17^. Goldschmidt’s algorithm is more amenable to parallelism and has the advantage of the absence of division over the original Newton–Raphson algorithm^1,18^. For these reasons, Goldschmidt’s algorithm is more suitable for hardware implementations^1^. Moreover, Bakhshali’s method provides faster convergence than the former two approaches, yet it involves higher computational complexity per iteration^19^.

The approximation methods are generally used when the true function is unknown or when the cost of the function is too high in terms of time or computational complexity. The approximation works well with smooth functions that have a continuous derivative. However, the derivative of the square root function rapidly changes as it approaches zero, imposing a computational challenge^1,20^. Hence, the use of approximation for the square root function is usually considered over a limited interval. Approximation methods using lookup tables (LUTs) are relatively fast, but their memory requirements increase exponentially as the precision increases^17^. Interpolation between points in the lookup tables could be used to reduce their size. Nonetheless, the increased computational time due to interpolation hinders the acceleration gained from using lookup tables. Similarly, employing approximation via high-order polynomials heavily depends on multiplication and requires memory for storing their coefficients^17^. Our main motivation for this work is computationally based where our objective is the development of a square root algorithm with high accuracy and low latency. By achieving this objective, we anticipate the use of the algorithm for various applications that span a wide spectrum of calculations from root mean squares (RMS) and vector norms to control systems of power electronics and signal processing techniques^1,21–26^.

This paper presents a novel seed-generation approach and a single-iteration approximation algorithm for the square root of unsigned numbers. The latter algorithm is based on geometrical construction of a plane square to solve the problem of the square root. In this research work, our contributions are outlined below:

- An initial estimation of the square root as a seed value, based on bit manipulation requiring only one addition operation and a single right shift, is described. The proposed seed yields significant reduction in the average number of iterations for Newton–Raphson’s and Bakhshali’s methods.
- A quadrature-based square rooting algorithm of higher accuracy compared to Newton–Raphson’s and Bakhshali’s methods.
- A lower latency, measured in the number of clock cycles, is achieved by our square rooting algorithm compared to Newton–Raphson’s and Bakhshali’s methods.

The rest of this paper is organized as follows: first, we review existing methods of the square root approximation and give their corresponding pseudocodes. The following section is devoted to elucidating and analyzing the proposed algorithms of seed generation and square root approximation. Then, the performance and accuracy results of the proposed square rooting algorithm are discussed and compared with other methods. Finally, we state our concluding remarks and directions for future work.

## Related work

In this section, we briefly describe some of the existing and well-known methods for calculating the square root value of an unsigned number *x*. Obtaining complex square roots of negative numbers is left out of the scope of this article. Before that, we review the fixed-point and floating-point representations of an *n*-bit number *x*.

### Fixed-point and floating-point formats

Fixed-point formats are conveniently used for signal processing such as audio and video streams^27^. Many signal processing systems adopt fixed-point formats because they offer reduced energy and power requirements as well as high processing speeds. Their use can be also beneficial by enabling better balancing in the datapath when utilizing integer arithmetic units^26,28^. For an *n*-bit fixed-point number *x*, the value of the number is given by:

1 $$\begin{aligned} x=2^z \sum \limits _{i=0}^{n-1}{b_i 2^{i-n}}, \end{aligned}$$

where *z* indicates the position of the binary point from the most significant bit while $b_i$ represents the bit chain of *x*. An alternative way of representing fixed-point numbers is by using a scale factor^27^. For example, a 16-bit signed binary format with a scale factor of $2^{-5}$ can represent numbers as small as $-1024.00000$ and as large as $+1023.96875$. The scale factor of fixed-point binary numbers is typically a power-of-two value. This is convenient as the scaling process is simply carried out using a few shift operations.

For applications where accuracy matters more than speed or in situations where numbers could be very small or very large, floating-point arithmetic is essential^29^. This is due to the fact that floating-point formats provide tradeoffs between range and precision. A floating-point number *x* consists of three parts: the sign, the mantissa, and the exponent. Typically, *x* can be represented as below:

2 $$\begin{aligned} x=(-1)^{S_x } \times 2^{E_x} \times 1.F_x, \end{aligned}$$

where $S_x$ is the value of the sign bit, $E_x$ is a biased integer value, and $F_x$ is the fraction part of the mantissa. We assume heretofore that $x \ge 0$. By taking the square root of *x*, it follows that:

3 $$\begin{aligned} \sqrt{x} = \sqrt{2^{E_x} \times 1.F_x}. \end{aligned}$$

There are two possibilities to express the right-hand term of Eq. (3):

4 $$\begin{aligned} \sqrt{2^{E_x} \times 1.F_x} = {\left\{ \begin{array}{ll} 2^{\lfloor E_x /2 \rfloor } \times \sqrt{1.F_x}, &{}\quad \text {if }~ E_x~\text { is even.} \\ 2^{\lfloor E_x /2 \rfloor } \times \sqrt{2 \times 1.F_x}, &{}\quad \text {if }~ E_x~\text { is odd.} \end{array}\right. } \end{aligned}$$

Therefore, computing the square root of an unsigned floating-point number *x* is reduced to calculating its value over the interval [1, 4), regardless of whether the floating-point number is given in a single or double-precision format^11,30^.

In this study, we consider 24-bit unsigned integer numbers assuming the IEEE 754 standard of a 23-bit mantissa plus the hidden bit. The purpose of this is to allow for the conversion from a fixed-point to a floating-point format. The conversion of a number with a fixed-point format to its corresponding floating-point representation is further detailed in^27,30^.

### Nonrestoring method

The nonrestoring method is one of the most popular subtractive methods used in digital circuits for both division and square root operations^31^. The method has low convergence yet provides accurate results. The algorithm takes the input *x* as the radicand and iteratively calculates the square root *s* and the remainder *R* knowing that $x = s^2 + R$. The binary representation of the radicand is divided into two-bit groups starting from the binary point and towards both directions. If the number of bits is odd, an extra bit is appended, preserving the original value. The pair of bits 01 is subtracted from the first group starting from the most significant group. If the result is positive, then the first digit of the quotient is equal to 1; otherwise, it is equal to 0. For each iteration, we pull the next group of bits and subtract $s_i 01$ if the remainder is positive or add $s_i 11$ if the remainder is negative. This process repeats until the end of all pairs^10^. The pseudocode for computing the square root using the nonrestoring method is provided in Algorithm (1)^32^.
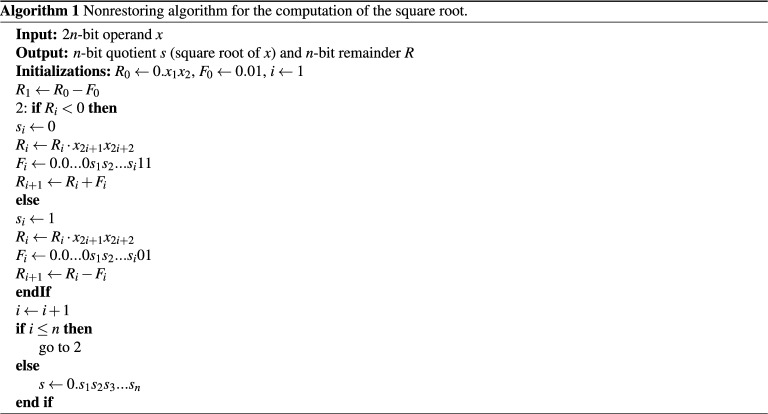


### Newton–Raphson’s method

Newton–Raphson is a popular and simple iterative method where an initial estimation is refined progressively until the desired precision is reached^2^. It has a quadratic convergence, which means that the accuracy of the resulting square root doubles at each iteration^16^. Generally, such iterative methods require an initial guess as a seed to the algorithm. This seed may be a positive number between 1 and *x* and can be selected in a way to yield fewer iterations. The seed value can be obtained via a variety of approaches, including the use of lookup tables^33,34^, polynomial approximation^35^, or a combination of both, called piecewise polynomials^36–38^. The seed can also be generated by faster means of bit-manipulation techniques^39^ and the selection of a magic constant^40–42^. In addition, a nonstandard method by Schwarz and Flynn in the form of Partial Product Array (PPA) is available^43^. The method uses direct binary multiplication, which can be implemented using logical AND gates that sum to an approximation of the square root.

The original Newton–Raphson formula recursively computes the square root value $s_i$, as given below:

5 $$\begin{aligned} s_{i+1}=0.5 \left( s_i + \frac{x}{s_i} \right) , \quad \text {for } i \ge 0, \end{aligned}$$

with $s_0$ being equal to the seed value. To avoid division by $s_i$ in each iteration, the inverse of the square root is used as an initial guess. The resulting value of the final iteration is then multiplied by *x* to generate the square root value^44^. Thus, the corresponding iterative equation to compute the inverse of the square root $r_i$ can be formulated as follows:

6 $$\begin{aligned} r_{i+1} = 0.5 r_i \left( 3-r_i^2 x \right) , \quad \text {for } i \ge 0. \end{aligned}$$

It follows that this method requires three multiplications, a subtraction from a constant, and a one-bit right shift in each iteration, followed by a final multiplication operation by *x* to obtain the square root value. In terms of hardware execution, the Newton–Raphson method serializes arithmetic operations in a single pipeline leading to a constrained throughput within the datapath^18^. The corresponding pseudocodes of the two Newton–Raphson methods used to calculate the square root based on Eqs. (5) and (6) are provided below in Algorithms (2) and (3), respectively.
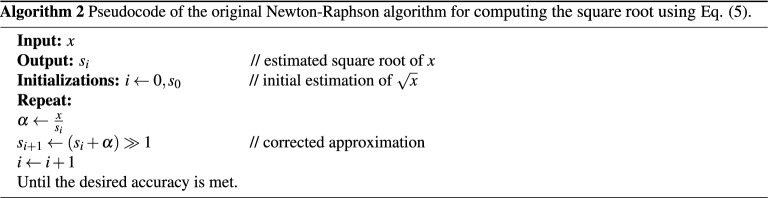

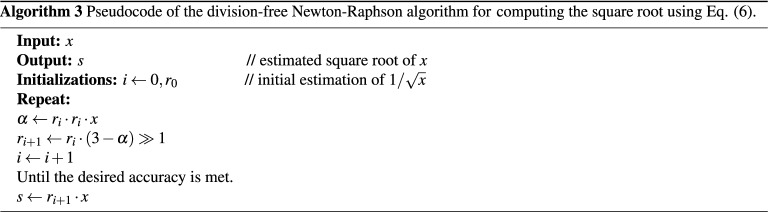


### Goldschmidt’s method

Another iterative method to calculate the square root was developed by Goldschmidt^45^. This method yields better performance compared to the Newton–Raphson as it enhances the utilization of the pipeline because of reduced data hazards. It also avoids the final multiplication to obtain the square root value. An interesting fact about Goldschmidt’s method is that it is more suitable for implementation in hardware than software^2^. The method is derived from a Taylor series expansion of the square root of *x* given as $\sqrt{s^2+R}$, where *s* is the square root and *R* is the remainder. This method computes both the square root and its inverse, starting with the seed value $s_0$ as an initial estimate to $1/\sqrt{x}$. The goal is to find a series of *n* instances of $s_i$, which makes the product ($x \times s_0^2 \times s_1^2 \times \cdots \times s_{n-1}^2$) equals 1. Then, the product ($s_0 \times s_1 \times \cdots \times s_{n-1}$) would approach $1/\sqrt{x}$. Therefore, ($x \times s_0 \times s_1 \times \cdots s_{n-1}$) would be equal to $\sqrt{x}$^46^. Note that to obtain the inverse of the square root from the described algorithm, the final value of the listed $h_i$ needs to be doubled. The pseudocode of Goldschmidt’s method is given in Algorithm (4)^1^.
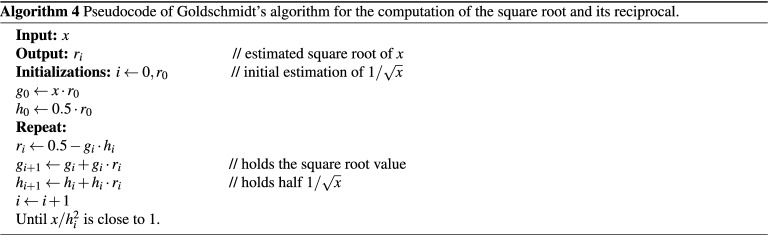


### Bakhshali’s method

This is a method for approximating the square root of a number that was first reported in an ancient Indian mathematical document known as the Bakhshali manuscript^19^. Given the initial estimate of the square root value $s_0$, the equivalent modern equation of Bakhshali’s method is as follows:

7 $$\begin{aligned} s_{i+1} = s_i + q_i - \frac{q_i^2}{2 \left( s_i + q_i \right) }, \end{aligned}$$

where $q_i = (x - s_i^2) / (2 s_i )$. The accuracy of the resulting square root converges faster than Newton–Raphson, i.e., quadruples at each iteration^47^. However, the method has higher computational complexity than Newton–Raphson. The pseudocode of Bakhshali’s method is disclosed in Algorithm (5).
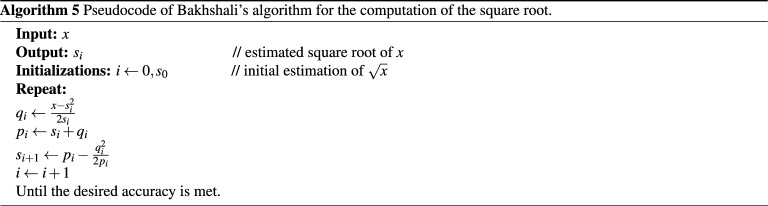


### Polynomial approximation method

Approximation by a real function such as polynomials is widely used due to its simplicity. It can be adjusted to yield the desired accuracy by controlling the number of polynomial coefficients^25,48^. Besides, the polynomial approximation method can be used for estimating the seed value of the square root for iterative algorithms^49,50^. The closer the value to the exact root, the fewer iterations are needed. Because the derivative of the square root function exhibits fast changes as it approaches zero, the square root value can only be approximated within a small interval to mitigate potentially high approximation errors^1^. Using a polynomial approximation, a square root function is implemented for Digital Signal Processors (DSP)^51^ for input values of *x*, where $0.5 \le x \le 1$. It is expressed in the following way:

8 $$\begin{aligned} s = {0.2075806} + 1.454895x - 1.34491x^2 + 1.10681x^3 - 0.536499x^4 + 0.1121216x^5. \end{aligned}$$

The input value is scaled to fall within the above-indicated interval. Then, the resulting value is scaled back after successfully approximating the square root value. The pseudocode of the polynomial approximating the square root value of *x*, given by Eq. (8), is described in Algorithm (6).
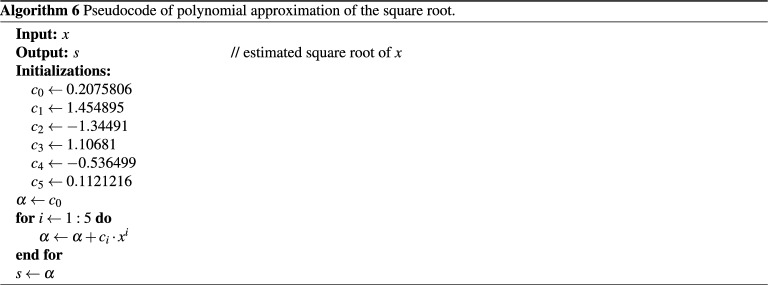


### Method by Dianov et al.

A recent square rooting method used for control systems of power electronics was developed by Dianov et al.^52^. The method is based on a division-free approximation of the parabola with a hyperbola. The achieved maximum relative error is 0.0050 which is acceptable for the majority of control systems of power electronics. The method is reported to be 1.6 times faster than the Newton–Raphson. The equation used to calculate the square root value is formulated as follows:

9 $$\begin{aligned} s=-0.039540 \cdot \frac{x^2}{2^{3n}} + 0.526010 \cdot \frac{x}{2^n} + 0.518555 \cdot 2^n, \end{aligned}$$

where *n* satisfies the condition $2^n \le s < 2^{n+1}$. The pseudocode of Eq. (9) is given in Algorithm (7).
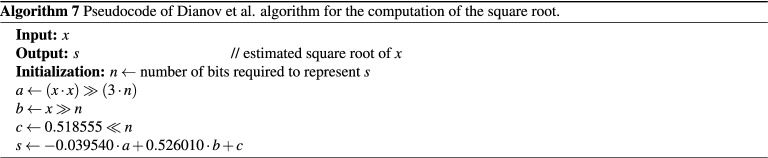


### Blinn’s method

Blinn’s method^53^ is a bit-manipulation technique that approximates the square root of *x*, represented in a single-precision format, using one addition and a single right shift. The method gives a rough approximation of the square root value and may be used to generate “good seeds for iterative refinement techniques”^53^, p. 130. It is well-known that for any positive 32-bit floating-point number float(*x*), the decimal value of *x* is obtained using the following formula:

10 $$\begin{aligned} x =\left( 1+F_x \cdot 2^{-23} \right) \times 2^{E_x-127}, \end{aligned}$$

where $F_x$ corresponds to the mantissa part and $E_x$ is the biased exponent of float(*x*). Computing the square root *s* using Blinn’s equation can be formulated as follows^48^:

11 $$\begin{aligned} s= {\left\{ \begin{array}{ll} \left( 1 + \frac{F_x \cdot 2^{-23}}{2}\right) \times 2^{(E_x - 127)/2}, &{}\quad \text {if}~ E_x~ \text {is odd.} \\ \left( \frac{3}{2} + \frac{F_x \cdot 2^{-23}}{2}\right) \times 2^{(E_x - 127-1)/2}, &{}\quad \text {if}~ E_x~\text { is even.} \end{array}\right. } \end{aligned}$$

In addition, if we let $B_x$ represent the bit-chain of the two’s complement of float(*x*), then the square root $B_s$ of $B_x$ is obtained using the following formula:

12 $$\begin{aligned} B_s = \left\lfloor \frac{B_x}{2} \right\rfloor + 127 \times 2^{22}. \end{aligned}$$

In this case, the division by 2 is performed first to guard against integer overflow. Then, the addition operation is carried out to obtain the approximated square root value^39^. The maximum relative error of this approximation is found to be 0.0607 and the approximated value is always larger than the exact square root value^48^. This relative error can be reduced to 0.03476 by replacing the quantity $127 \times 2^{22} = 532,676,608$ in Eq. (12), by a smaller value that is equal to 532,369,100^48^. The pseudocode of Blinn’s algorithm based on Eq. (11) is provided in Algorithm (8).
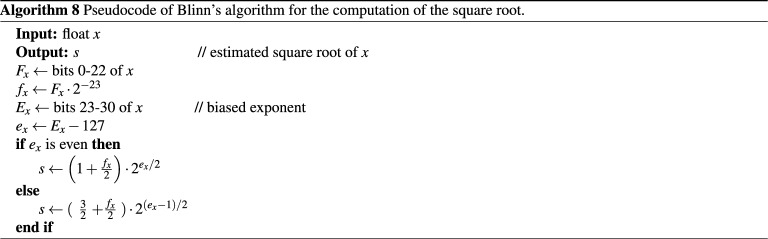


## Proposed algorithms for seed generation and square rooting

In this section, we describe a low-complexity approach for the initial estimation of the square root value. This approach is based on bit-manipulation techniques and uses only one addition and a single-bit right shift. Next, a quadrature-based square rooting algorithm is proposed employing a single-cycle lookup table. The algorithm is based on the geometrical construction of a square from a plane rectangle to solve the problem of the square root. The proposed initial estimation is employed as a seed in the quadrature process to help shape the dimensions of the plane rectangle and to avoid number factorization.

### Seed generation based on bit manipulation

For any power-of-two positive square integer *x* that is written as $2^{n-1}$ where $n \in {{\mathbb {N}}}$ and $n \ge 1$, we can immediately obtain its square root by discarding the least significant half of its binary representation $x_{bin}$. This is equivalent to right shifting $x_{bin}$ by $\lfloor n/2 \rfloor$ bits. For example, the square root of $2^6 = 64_{10} = 1000000_2$, where *n* equals 7, is $1000_2$ $= 2^{\lfloor n/2 \rfloor }$ after right shifting the three least significant bits ($3= \lfloor 7/2 \rfloor$). Note that *n* here is equal to $\lfloor \log _2 x \rfloor + 1$, which corresponds to the minimum number of bits required to represent *x*.

By extending the above procedure to numbers that are not powers of two yet have an integer square root, we can improve the initial estimation of the square root, $s_0$, by averaging the integer value of the most significant half (MSH) of $x_{bin}$ and the quantity $2^{\lfloor n / 2 \rfloor }$. For instance, the square root of $25_{10} = 11001_2$ can be obtained by averaging the most significant half of the sequence, $110_2 = 6_{10}$, and the quantity $2^{\lfloor 5/2 \rfloor } = 4_{10}$. The obtained value is equal to $(6+4)/2 = 5$, which is the correct square root value of $25_{10}$. Therefore, we obtain the following expression for $s_0$:

13 $$\begin{aligned} s_0 = 0.5 \times \left( MSH + 2^{\lfloor n / 2 \rfloor } \right) . \end{aligned}$$

Clearly, we can replace dividing the numerator by 2 in Eq. (13) by performing a single-bit right shift operation. Table 1 shows a list of square numbers with their square roots estimated with values of $s_0$ that are obtained by applying the aforementioned averaging step.

**Table 1:** Initial estimations of $s_0$ for selected integer square numbers.

Square number (*x*)	${x}_{{bin}}$	${0.5 \times } \left( {MSH + 2}^{{\lfloor n/2 \rfloor }}\right)$	${s_0}$	Exact square root $(\sqrt{{x}})$
9	1001^a^	$0.5 \times (2 + 4)$	3	3
25	11001	$0.5 \times (6 + 4)$	5	5
100	1100100	$0.5 \times (12 + 8)$	10	10
289	100100001	$0.5 \times (18 + 16)$	17	17
361	101101001	$0.5 \times (22 + 16)$	19	19
529	1000010001	$0.5 \times (16 + 32)$	24	23
841	1101001001	$0.5 \times (26 + 32)$	29	29
3969	111110000001	$0.5 \times (62 + 64)$	63	63
5329	1010011010001	$0.5 \times (83 + 64)$	73	73
16129	11111100000001	$0.5 \times (126 + 128)$	127	127
17424	100010000010000	$0.5 \times (136 + 128)$	132	132
28561	110111110010001	$0.5 \times (223 + 128)$	175	169
90601	10110001101010001	$0.5 \times (355 + 256)$	304	301
186624	101101100100000000	$0.5 \times (364 + 512)$	438	432
67059721	11111111110100000000001001	$0.5 \times (8186+ 8192)$	8189	8189
1073807361	1000000000000010000000000000001	$0.5 \times (32,770+ 32,768)$	32,769	32,769

$^{\textrm{a}}$ The underlined half of the binary representation indicates the most significant half (MSH) of $x_{bin}$.

**Figure 1:**
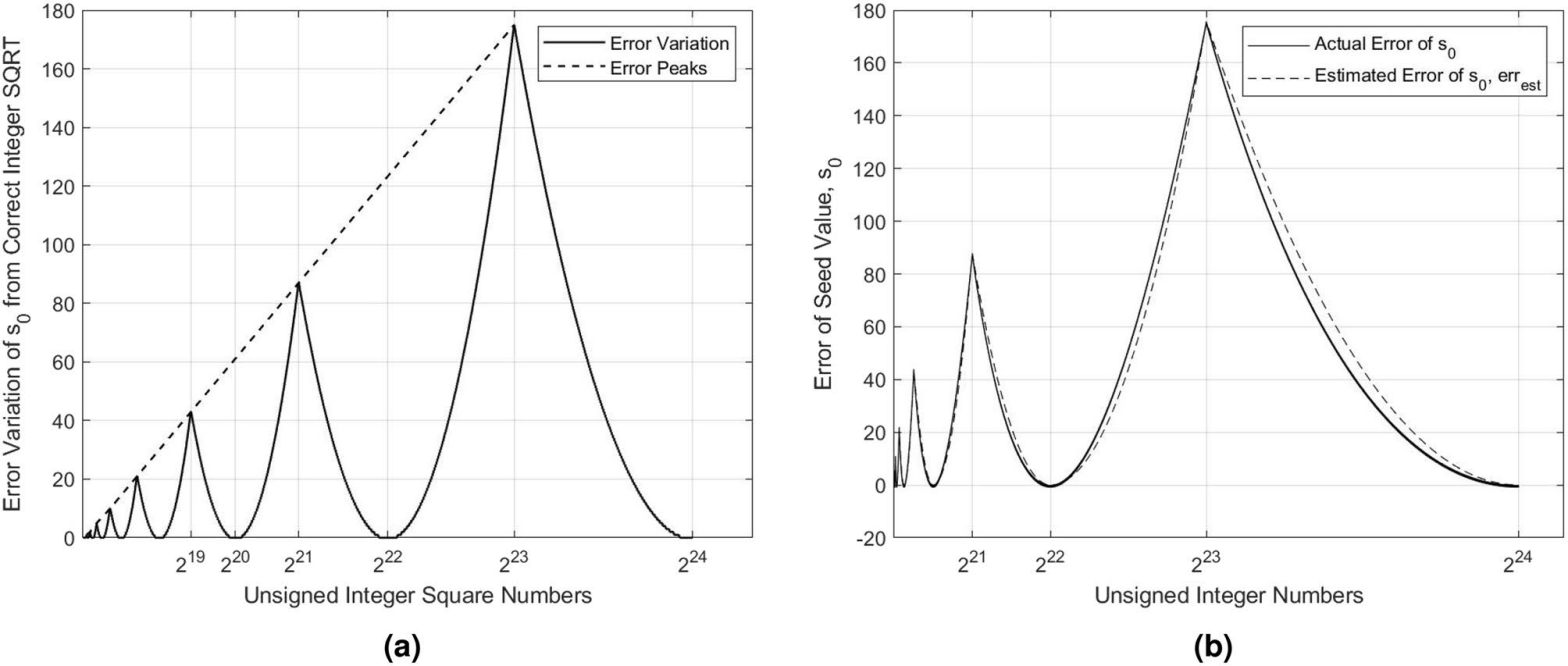
Generated error from seed estimation $s_0$: (**a**) A scaled plot showing the pattern of the error of $s_0$, as it varies from the correct integer square root. Peaks of the error increase linearly, whereas the shape between consecutive error peaks forms a parabola-like curve (PLC). (**b**) Variations of the actual and estimated errors of the generated seed value, $s_0$.

As shown in the mentioned table, this procedure does not always yield an exact square root. For example, for $x=529=23^2$, the estimated value of the square root is 24. We begin to observe a deviation from the correct integer square root when *n* equals 9 bits. In Table S1, provided in Appendix A due to its length, we disclose the first three occurrences of these deviations. The maximum deviation is consistently found in the middle of any given batch of deviated seed values of $s_0$. As depicted in Fig. 1a, the local maxima of these deviations increase linearly. The errors between each consecutive pair of local maxima (or peaks) form a pattern of connected Parabola-Like Curves (PLC). We use the term “parabola-like” to indicate that the two sides of a PLC are asymmetric, having two different focal points. We also observe in Fig. 1a that the vertex of the PLC is always located at the even powers of two. The error value at a vertex is equal to zero since this point always represents a square number. In contrast, the maximum deviation occurs at the odd powers of two. Fig. 1a instantiates the occurrence of these maximum deviations at $x = 2^{19}, x = 2^{21}$, and $x = 2^{23}$. Since the PLC curve is asymmetric, the two maxima can be employed to find the focal point for each side of the curve with respect to a common vertex. In this regard, we make the following two observations:

- For points between two adjacent vertices, they share the same peak.
- For points between two adjacent peaks, they share the same vertex.

We note that the initial estimation, given by $s_0$ in Eq. (13), could be used as a seed value for other iterative square rooting algorithms such as Newton–Raphson and Bakhshali’s methods. The pseudocode for the algorithm used to calculate the initial estimation of the square root, $s_0$, is given in Algorithm (9).
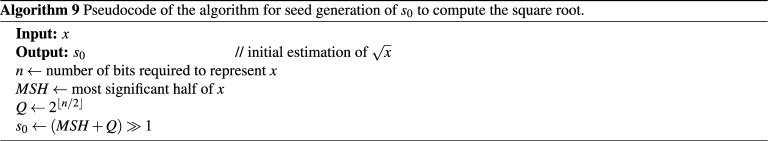


#### Error analysis of the generated seed

We define the error, $err_{est}$, to be equal to the difference between the initial guess $s_0$ and the correct square root value. Let $y_{peak}$ denote the local maximum error and $x_{peak}$ define the value of the *x* location that corresponds to $y_{peak}$. By plotting the approximation error as a function of $x_{peak}$, the obtained fitting results, using the MATLAB Curve Fitting Tool, generate the following first-degree polynomial for the corresponding $y_{peak}$:

14 $$\begin{aligned} y_{peak} = 0.06067 \cdot x_{peak} - 0.796. \end{aligned}$$

Next, we utilize $x_{peak}$ and $y_{peak}$ to calculate the focal point *p* for each side of the PLC, given its vertex *v*, as follows:

15 $$\begin{aligned} p = \frac{ \left( x_{peak} - v \right) ^2}{4 \times y_{peak}}. \end{aligned}$$

For $1 \le x < 2^{24}$, there are only eight peaks, which means that there are 16 different values of *p* to be computed for the entire error curve. Further, the error of any positive number *x* between the vertex *v* and $x_{peak}$ can be estimated using the following equation for the vertical parabola, given by:

16 $$\begin{aligned} err_{est} = \frac{\left( x-v \right) ^2}{4 \times p}. \end{aligned}$$

As depicted in Fig. 1b, the resulting $err_{est}$ (shown in a dashed line) provides a good estimation of the error produced by the seed value $s_0$.

### Quadrature-based square rooting algorithm

#### Background

The interest in the quadrature problem goes back to the time of ancient Greeks. It involves squaring a plane figure for the purpose of finding its area. This is achieved by geometrically constructing a square of the same area, hence the name “quadrature.” In particular, the quadrature of a rectangle is first documented as Proposition 14 of Book II of Euclid’s Elements^54^. In the context of differential equations, the term quadrature is currently employed to mean solving an equation in terms of integrals. The theory behind the quadrature of a plane rectangle is briefly described next.

**Figure 2:**
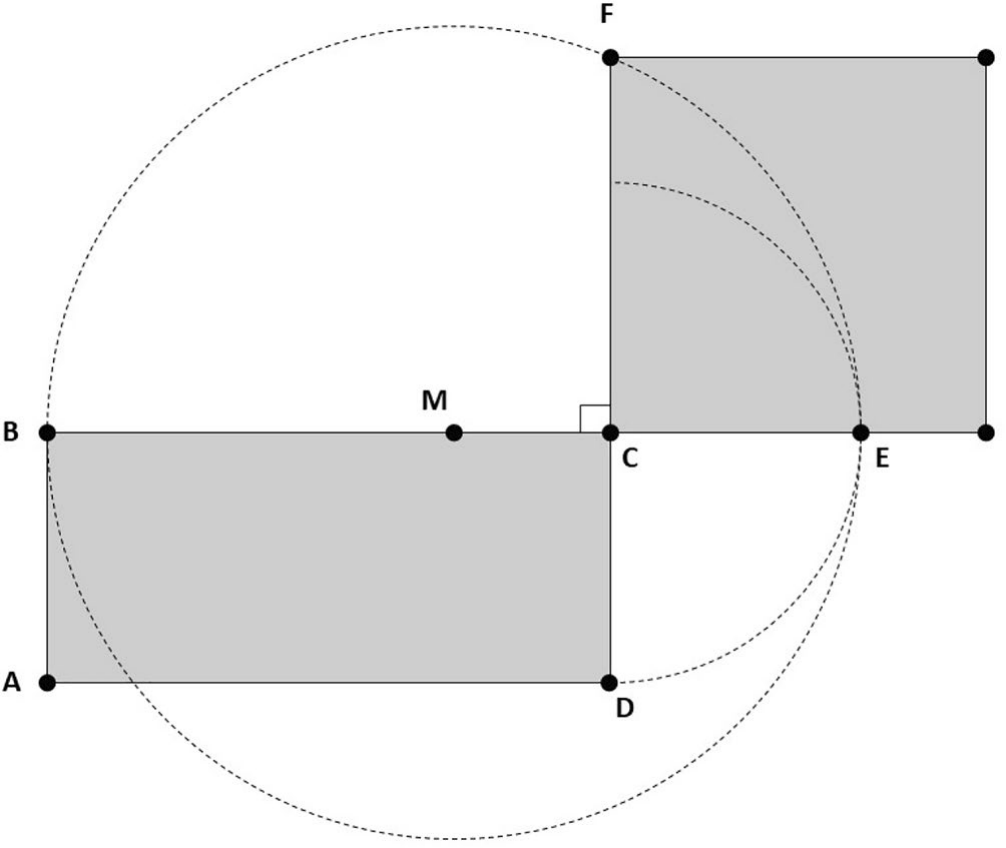
The quadrature of a plane rectangle $\sqsubset \! \sqsupset ABCD$.

Let $\sqsubset \! \sqsupset ABCD$ be an arbitrary rectangle (see Fig. 2). We extend the segment *BC* to the right and mark a new point *E*, where both *E* and *D* are at the same distance from *C*. Next, we bisect segment *BE* at the point *M*, which becomes the center of a circle with a radius of length *BM*. Then, we extend a line from point *C* perpendicular to segment *BE*. The intersection of this line with the circumference of the circle produces the segment *CF*. This represents one side of the desired square.

We can utilize this theory to computationally find an efficient solution to calculate the square root value. By connecting the two points *M* and *F* in the figure, we have a right triangle at the angle $\angle MCF$. Note that the hypotenuse of the triangle, given by segment *FM*, is equal to the radius of the circle. The lengths of both segments of this right triangle, namely segments *FM* and *CM*, are known values. The problem is reduced to finding the length of the segment *CF*, which corresponds to the desired square root value. Indeed, the use of the Pythagorean theorem requires solving for the square root. Therefore, by using basic trigonometry we derive the following set of equations to solve the above problem:

$$\begin{aligned} \sin \theta&= {\frac{{{\text {Opposite}}}}{{{\text {Hypotenuse}}}}} = {\frac{ {\overline{CM}}}{ {\overline{FM}}}}, \\ \theta&= \sin ^{-1} {\frac{{\overline{CM}}}{{\overline{FM}}}}, \\ \cos \theta&= {\frac{{{\text {Adjacent}}}}{{{\text {Hypotenuse}}}}} = {\frac{{\overline{CF}}}{{\overline{FM}}}}, \quad {\text {and}} \\ {\overline{CF}}&= \cos \theta \times {\overline{FM}}, \end{aligned}$$

where $\theta$ corresponds to the angle $\angle MFC$.

According to the instruction tables for different processor architectures, given in^55^, the floating point sine and cosine instructions cost 90–100 clock cycles per instruction for AMD K7, and 60–100 clock cycles for Intel Pentium. Further, fixed-point sine and cosine functions can be computed using the COordinate Rotation DIgital Computer (CORDIC) method. This is a well-known method based on iterative shift and add operations^56–58^. Trigonometric functions can also be approximated using linear interpolation utilizing lookup tables^59^. According to the MATLAB documentation^60^, fixed-point CORDIC costs one table lookup, two shifts, and three additions per iteration. On the other hand, linear interpolation costs two table lookups, one multiplication, and two additions. Our goal is to directly access the value of the desired trigonometric function in a single clock cycle utilizing a small-sized lookup table. Direct addressing into a lookup table can locate any entry in O(1) time^61^. Nonetheless, smaller lookup tables are preferable for area and power considerations. The value obtained from the lookup table is then used to calculate the estimated square root based on the quadrature method.

#### Implementation

Let *x* be the area of the aforementioned rectangle. Generally, we need to factorize *x* to obtain the two sides of the rectangle. However, we can avoid number factorization by taking the initial seed, $s_0$, as one side of the rectangle. The other side is simply obtained by dividing *x* over $s_0$. The average of both sides of the rectangle produces the hypotenuse of the right triangle *MCF*. Also, the difference between $s_0$ and the generated hypotenuse results in the opposite side of the angle $\theta$. Given the values of both the hypotenuse and the opposite segments of this right triangle, we can calculate $\sin \theta$ from which we can obtain the angle $\theta$.

**Figure 3:**
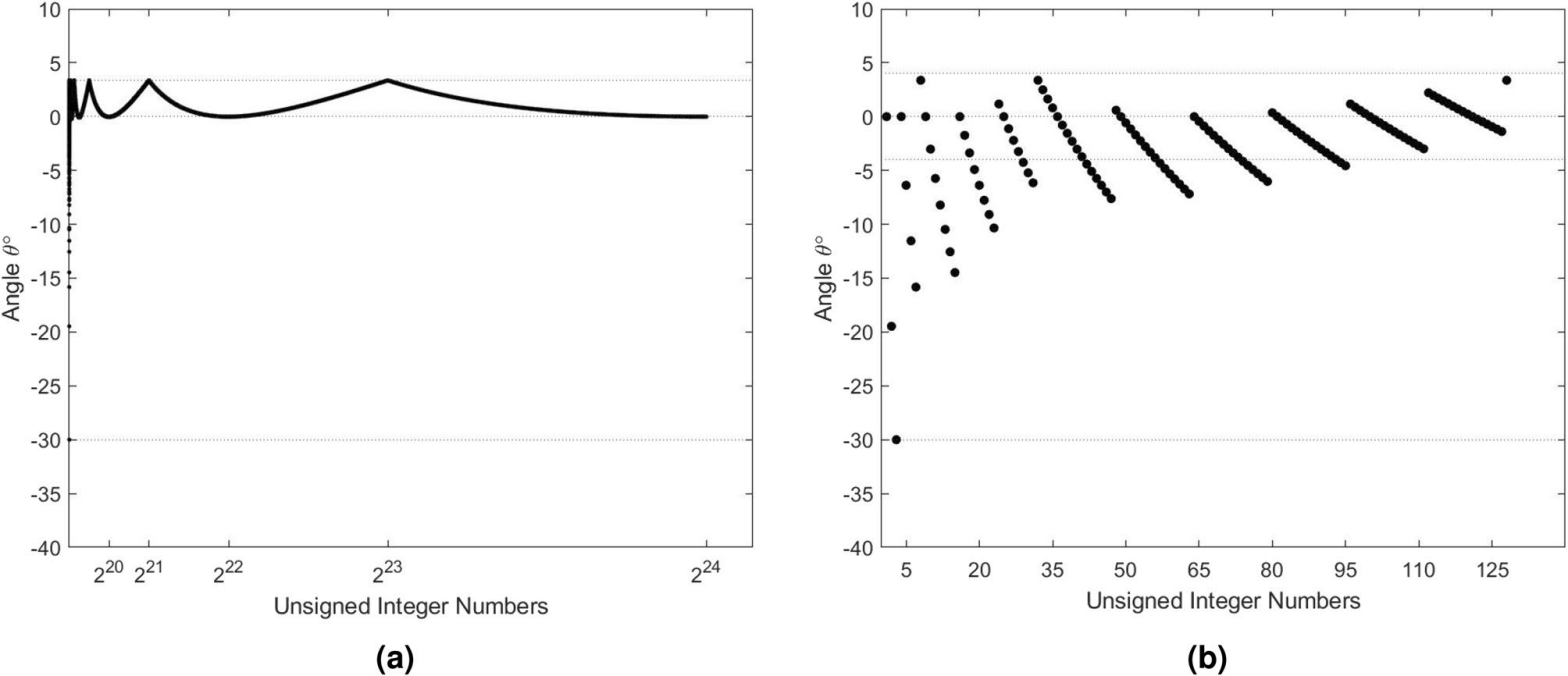
The range of $\theta$ (in degrees) as a function of *x*: (**a**) For all *x* up to $2^{24}-1$. (**b**) Zoomed plot for *x* up to the first 128 values. The first 95 values have $\theta \in [-30^\circ , 4^\circ )$ while all remaining values have $\theta \in (-4^\circ , 4^\circ )$.

The angle $\theta$ is equal to zero when the hypotenuse and the adjacent segment *CF* are coincident. This means that the shape we started with is a perfect square and $s_0$ is an accurate estimate of the square root. Less accuracy of $s_0$ translates to wider deviation of $\theta$ from 0$^\circ$. The worst case scenario is when the hypotenuse is almost perpendicular to the adjacent side with an angle of nearly $\pm 90 ^\circ$. Simulation results for all *x* values up to $2^{24}-1$ yield $\theta \in [-30^\circ , 4^\circ )$, when assigning $s_0$ to the width of the rectangle. Figure 3a shows a plot of the angle $\theta$ in degrees as a function of *x*. As illustrated in the figure, the angle $\theta$ is in the range (-4$^\circ$, 4$^\circ$) for all *x*, except for the first 95 values. The latter number is equivalent to only $0.05662\%$ of all *x* values, which is equal to $95 / 2^{24}$. Figure 3b displays the angle $\theta$ for the first 128 values of *x*. It shows that among these values, the first 95 have $\theta \in [-30^\circ , 4^\circ )$. Thus, the use of $s_0$ allows for the plane shape to be closer to a square, which results in $\theta$ being closer to zero.

**Figure 4:**
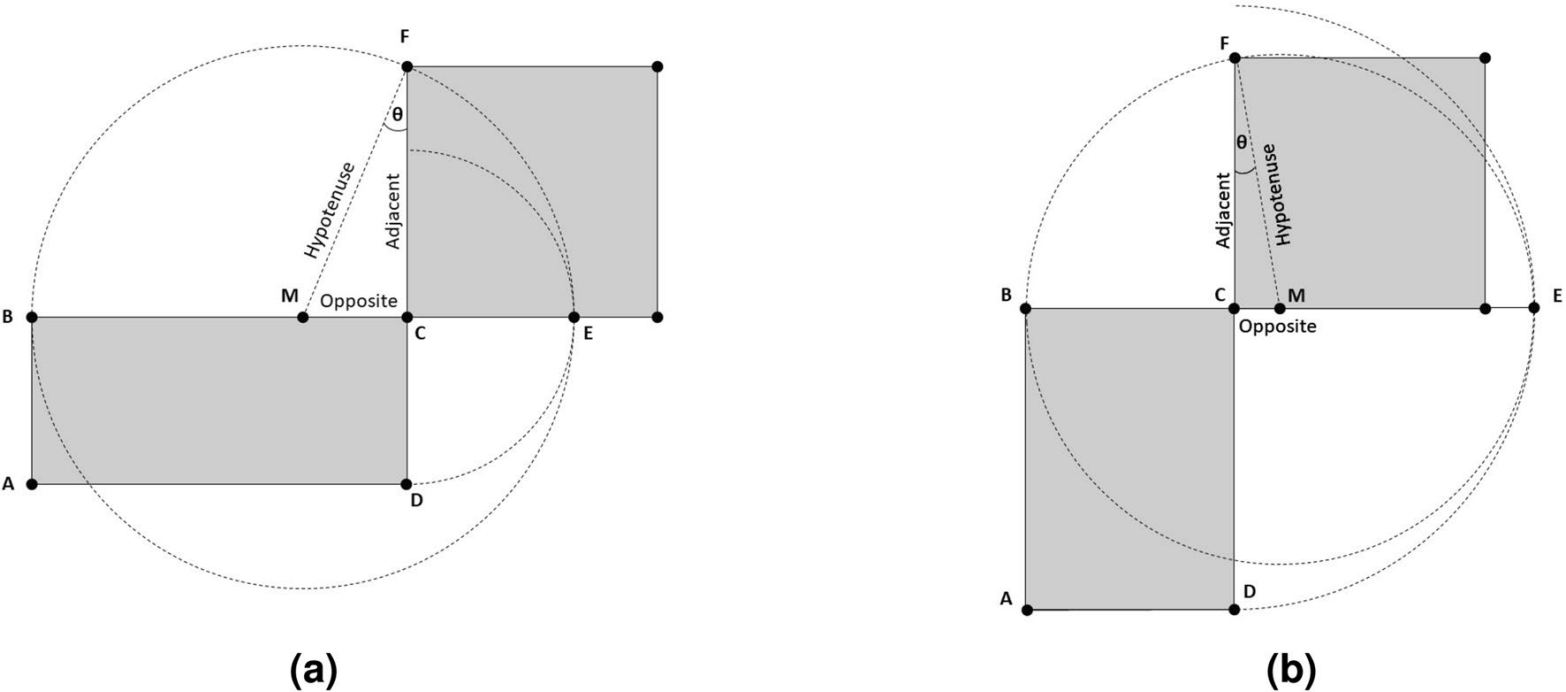
The quadrature of a plane rectangle: (**a**) A landscape rectangle produces positive values of $\theta$. (**b**) A portrait rectangle produces negative values of $\theta$.

The negative angle $\theta$ results from the orientation of the plane rectangle. For a portrait rectangle, the radius is larger than the width of the rectangle leading to a negative sign for the opposite segment. As a result, a negative value of $\sin \theta$ is produced. Given that $- \sin \theta$ is equal to $\sin (- \theta )$, the resulting angle $\theta$ is now negative. On the other hand, a landscape rectangle yields a positive $\theta$ as the calculated opposite segment remains positive. Figure 4 illustrates the impact of the shape orientation on the angle $\theta$. Our simulations also show that $s_0$ is greater than or equal to the correct square root of *x* for $93.86\%$ of all values (15,746,886 out of $2^{24}$). Based on this, the orientation of the corresponding rectangle is mostly landscape. Consequently, $\theta$ is positive for the majority of *x* values as depicted in Fig. 3a.

Next, we need to construct a lookup table of $\cos \theta$ values. This table is to be directly addressed by scaled values of $\sin \theta$. These scaled values determine the length of the lookup table. Since both $\sin \theta$ and $\sin (- \theta )$ map to the same cosine value, we need only consider one polarity of $\theta$. As a result, the range is reduced to the interval [0$^\circ$, 30$^\circ$] whose absolute values of $\sin \theta$ belong to the interval [0, 0.5]. A step size of 0.01 for this interval produces $(0.5 / 0.01) + 1 = 51$ values of $\sin \theta$ that can be mapped to corresponding 51 values of $\cos \theta$. Further reduction in the step size to 0.001 translates to 501 values of $\sin \theta$. The impact of the step size of $\sin \theta$ values on the size of the lookup table is presented in Table 2. We note here that the lookup table size is calculated based on using a 16-bit precision for each of the corresponding cosine values. For example, for a step size of 0.01, we need 51 cosine values. This translates to a LUT size of $51 \times 16$ bits $= 51 \times 2$ bytes $= 102$ bytes. The reported LUT sizes in the table are all given as the nearest power-of-two values to the actual sizes in bytes. In this case, the nearest power-of-two value to 102 is 128. The table also shows the utilization of the lookup table calculated as the ratio of the accessed entries to the total number of entries in the lookup table. The table shows that decrementing the step size of $\sin \theta$ leads to increases in the number of samples of $\sin \theta$, the size of the lookup table, and the number of accessed entries. However, it engendered a decrease in the LUT utilization. Based on the computed utilization values, it may be more efficient to maintain a LUT size of less than or equal to 1 KB.

**Table 2:** The impact of the step size of $\sin \theta$ on the size of the lookup table and its utilization, assuming a 16-bit precision of $\cos \theta$ values.

Step size of $\sin \theta$	Number of samples of $\sin \theta$	LUT size	Accessed entries	Utilization (%)
$1\times 10^{-2}$	51	128 B	24	37.50
$1 \times 10^{-3}$	501	1 KB	100	19.53
$1 \times 10^{-4}$	5001	16 KB	635	7.75
$1 \times 10^{-5}$	50,001	128 KB	5929	9.05
$1 \times 10^{-6}$	500,001	1 MB	58,870	11.23

#### Flowchart and pseudocode of the proposed algorithm

**Figure 5:**
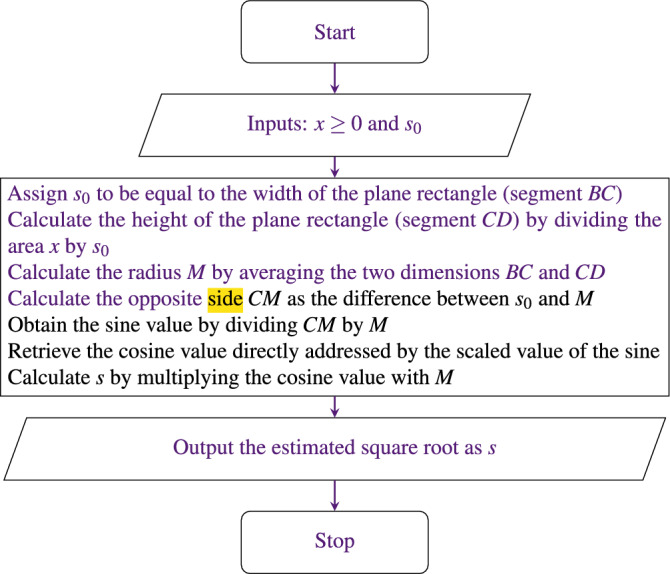
Flowchart of our proposed algorithm to compute the square root of an unsigned number *x*.

The pseudocode for approximating the square root value of *x*, denoted by *s*, takes both *x* and the seed value $s_0$ as inputs. The algorithm starts with finding the second dimension of the plane rectangle of area *x*, given that $s_0$ is equal to the first dimension. The average of these two dimensions corresponds to the radius of the guide circle, and also, to the hypotenuse of the resulting right triangle. The opposite side of this triangle is simply the difference between $s_0$ and the radius. By finding both the hypotenuse and the opposite sides, the sine value can be obtained. Next, we retrieve the corresponding cosine value utilizing a lookup table that is directly addressed by a scaled value of $\sin \theta$. Finally, the estimated square root *s* is obtained by multiplying the cosine value by the calculated hypotenuse. The flowchart of our proposed algorithm is displayed in Fig. 5 and its complete pseudocode is given in Algorithm (10). The naming of the variables in the pseudocode corresponds to the segments utilized in Fig. 2.
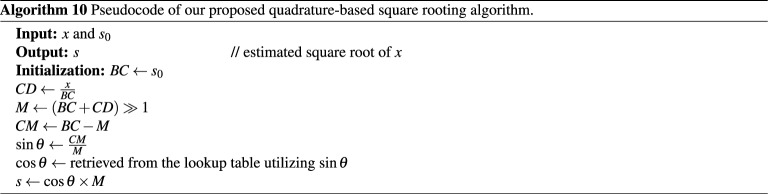

## Experimental results and discussion

Before we embark on discussing our experimental results, we present in Table 3 a summary of the advantages and disadvantages of the reviewed square rooting methods and our proposed algorithm. The table also provides a characterization of these methods and lists some of the corresponding references in the literature.

**Table 3:** Characteristics of selected square root methods and our proposed algorithm.

Square root method	Method type	Advantages	Disadvantages	References
Newton–Raphson	Iterative	Simple	Includes division operation	^1,15^
		Fast convergence (quadratic rate)	Requires initial estimation (seed value)	
			Latency and number of iterations depend on the quality of the chosen seed	
			Large numbers require extra iterations	
Goldschmidt	Iterative	Absence of division operation	Slow for multiplication using more than 16 bits	^1,18,46^
		Fast convergence (quadratic rate)	Truncates 64-bit numbers to 32 bits, producing calculation errors	
		Simultaneously calculates the square root and reciprocal square root	Requires initial estimation (seed value)	
		Suitable for hardware	Low performance in software implementations	
		Amenable for parallelism due to independent multiplication operations	Large numbers require extra iterations	
Blinn	Bit manipulation	Fast and simple	Rough approximation (high maximum relative error)	^48^
Bakhshali	Iterative	Very fast convergence (quadruple rate)	Contains division operation	^19^
		Avoid large numbers in the calculation of the square root	Requires initial values	
			Latency and number of iterations depend on the quality of the chosen initial values	
			Large numbers require extra iterations	
Polynomial approximation	Approximation by real function	Good speed (e.g., fast piecewise interpolation)	Difficult to achieve high accuracy	^15^
		Carried out efficiently in both software and hardware circuits	Requires extra memory for coefficients and interpolation points	
			Requires rescaling before and after the calculation of the square root.	
Dianov et al.	Approximation by real function (hyperbola)	Absence of division operation	Single iteration	^52^
			Difficult to achieve high accuracy	
Nonrestoring	Subtractive	Requires a limited number of arithmetic operations	High computational time	^4,15,48,62^
		Very simple, multiplication-free	Slow convergence (linear rate)	
		Suitable for hardware implementation	Arithmetic operations need to be performed at full-length	
		Exact value of the square root is obtained	Additions and subtractions using non-redundant digit sets negatively impact the amount of required hardware	
Proposed algorithm	Quadrature-based square rooting	Simple	Contains division operation	
		Single iteration		
		High accuracy can be achieved using a small-sized lookup table		

### Computing platform and experimental conditions

Our experimental results were obtained using the MATLAB computing environment R2020 installed on an Intel Core i7-10510U machine. This system has a Central Processing Unit (CPU) with a clock frequency of 2304 Mhz, 16.0 GBs of RAM, and running Microsoft Windows 11 as the operating system.

The performance and accuracy results of the reviewed methods are evaluated and compared to the proposed algorithm. The seed selection for iterative methods is based on the initial estimation reported in the works of Dianov et al.^1,52^. For Newton–Raphson’s method, the initial value is estimated by finding *k*, so that $s_0 \in [2^k, 2^{k+1})$. The midpoint of this interval is assigned to $s_0$. We opt to use the same seed for Bakhshali’s methods since both algorithms have similar properties. For Goldschmidt’s algorithm, the seed is estimated using a lookup table or special hardware such that $1/2 \le s_0^2 \cdot x \le 3/2$^46^. To simplify the problem, one side of the above inequality is selected as a seed. The selection of the lower limit ($s_0 = 1/\sqrt{2x}$) yields a maximum relative error of 0.0194. On the other hand, the selection of the upper limit ($s_0 = \sqrt{3/2x}$) allows for achieving results of higher accuracy. Accordingly, we chose to use the latter seed value since it would also results in the least relative error. Moreover, we consider herein Blinn’s method as a seed generation technique, for it gives a rough approximation of the square root value. It is included in the analysis of the impact of seed selection on the number of iteration for convergence methods.

### Performance evaluation and comparison

The computational complexity of the selected square rooting algorithms is analyzed in terms of the required number of clock cycles. First, the number of arithmetic and logical operations is tallied for each iteration of the algorithm. Then, this number is multiplied by the number of iterations required to generate the square root. This yields the total number of operations for each type of algorithm. Afterward, the latency of each operation type is obtained by multiplying the total number of operations with its corresponding number of clock cycles. Finally, we sum up all these latencies to obtain the overall number of clock cycles for each algorithm. The latency values for each arithmetic and logical operation are obtained from a published manual with the best-case latencies being considered^55^. According to this reference, it takes one clock cycle for addition and comparison, four cycles for the shift operation, two cycles for multiplication, and 37 cycles for division. We provide in Table 4 the total number of clock cycles for six square rooting methods in addition to our proposed algorithm. We note that the approximation method by Dianov et al.^52^ yields the least number of clock cycles with a value of 34 then Goldschmidt with 35 clock cycles followed by the polynomial approximation with 39 clock cycles. Whereas these performance results are obtained with reduced accuracy, the approximation by the aforementioned methods can still be acceptable for square root calculation when the latency is of higher importance. Our proposed algorithm comes next in rank with a total latency of 85 clock cycles. Finally, Newton–Raphson, followed by Bakhshali and nonrestoring methodcomplete the obtained ranking with 89, 92 and 313 clock cycles, respectively.

**Table 4:** Performance comparison of different square rooting methods based on the total number of clock cycles.

Square rooting method	Number of iterations	+, -, &, |, ${^{\hat{\,}}}$	${\ll , \gg }$	Compare	${\times }$	${\div }$	Clock cycles
Nonrestoring	12	60	60	13	0	0	313
Newton–Raphson	2	4	2	3	0	2	89
Bakhshali	1	4	2	2	2	2	92
Goldschmidt	2	8	2	3	8	0	35
Polynomial approximation	1	5	0	0	17	0	39
Dianov et al.	1	6	5	4	2	0	34
Proposed algorithm	1	2	1	0	2	2	85^a^

^a^A single cycle is added for the latency of the lookup table.

### Accuracy evaluation and comparison

In this section, we analyze the impact of using different sizes of the lookup table on the accuracy of the estimated square root. Next, the impact of using different seed generation techniques on the accuracy of the proposed square rooting algorithm is evaluated. Finally, we present and compare the accuracy results of six square-rooting methods with our proposed algorithm. For each method, we compute the maximum relative error (MRE) and the accuracy in bits^25,63^. The MRE value is calculated as follows:

17 $$\begin{aligned} {MRE} = max\left( \frac{|s - \sqrt{x}|}{\sqrt{x}} \right) . \end{aligned}$$

Based on the above, the accuracy (ACC) in bits is obtained by applying the following equation:

18 $$\begin{aligned} ACC = - \log _2 {MRE}. \end{aligned}$$

The smallest lookup table of 128 bytes allows for a maximum relative error of 0.0017 and an accuracy of slightly more than 9 bits. The use of larger lookup tables starts to reach a plateau at around 15 bits of accuracy, as indicated in Table 5. This is due to the limitation imposed by the precision of the stored cosine values at 16 bits. For instance, when we further extend the representation of the cosine value using double precision (64 bits), the step size of $1 \times 10^{-5}$ increases the accuracy from 14.2720 to 18.8321 resulting in absolute improvement of about 4.5 bits. Moreover, the impact of double precision on the improvement of the accuracy is reduced for smaller step sizes. For example, the accuracy increases to only 12.1903 bits (from 12.1711 bits) and to 15.5511 bits (from 14.0919 bits) for step sizes of $1 \times 10^{-3}$ and $1 \times 10^{-4}$, respectively. For applications of limited area and power requirements, a lookup table of up to 1 KB is sufficient^63^. An increase in the LUT size from 128 bytes to 1 KB results in an increase in the accuracy by three more bits while maintaining the same latency thanks to the constant time complexity resulting from its direct addressing. We note that decrementing the step size of $\sin \theta$ to $1 \times 10^{-7}$ and $1 \times 10^{-8}$, while maintaining a 16-bit precision of $\cos \theta$, would yield negligible improvements in the MRE and accuracy values. In fact, the obtained MRE values are $5.0086 \times 10^{-5}$ and $5.0082 \times 10^{-5}$ while their corresponding accuracy values are 14.2852 bits and 14.2854 bits, respectively.

**Table 5:** The impact of the step size of $\sin \theta$ on the accuracy results of our proposed square root algorithm, assuming a 16-bit precision of $\cos \theta$ values.

Step size of $\sin \theta$	LUT size	MRE	Accuracy (bits)
$1 \times 10^{-2}$	128 B	0.0017	9.2287
$1 \times 10^{-3}$	1 KB	$2.1683 \times 10^{-4}$	12.1711
$1 \times 10^{-4}$	16 KB	$5.7269 \times 10^{-5}$	14.0919
$1 \times 10^{-5}$	128 KB	$5.0547 \times 10^{-5}$	14.2720
$1 \times 10^{-6}$	1MB	$5.0130 \times 10^{-5}$	14.2840

**Figure 6:**
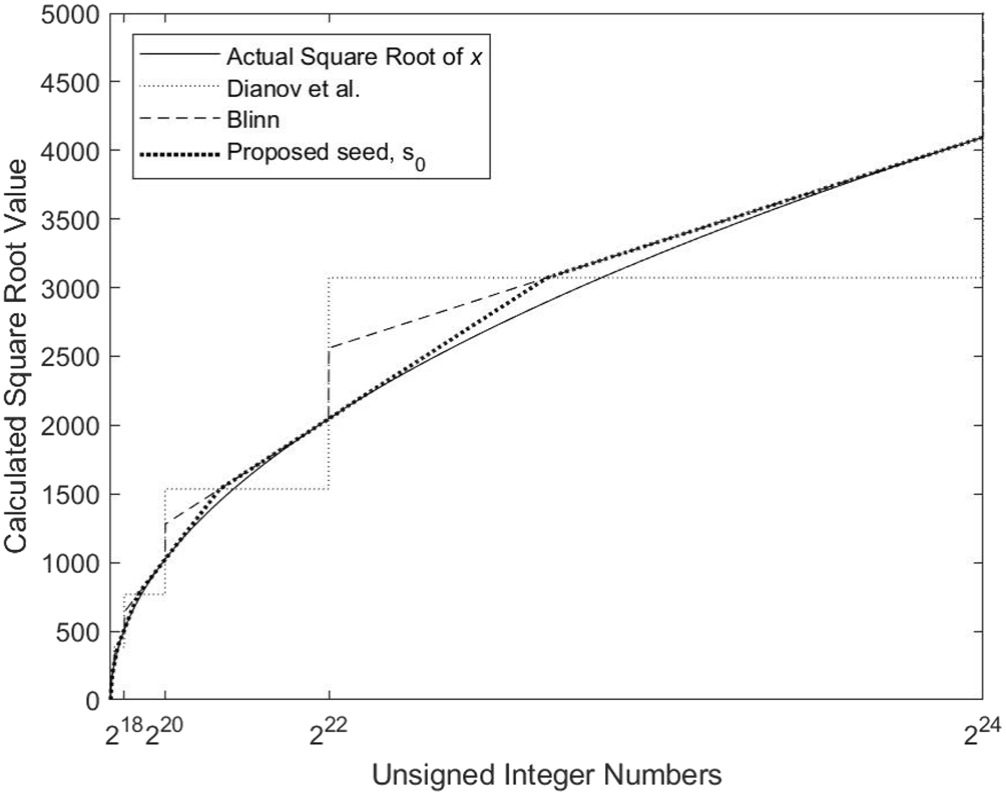
Comparison of the deviations of our proposed seed $s_0$, the reported seed $3 \times 2^{n-1}$ by Dianov et al., and Blinn’s method for seed generation from the correct square root values.

The impact of using different seeding techniques on the accuracy of the quadrature-based square rooting method is analyzed. Overall, the seeds: $3 \times 2^{k-1}$, Blinn, and $s_0$, are employed in the calculations of these results. For the first seed, $k = \lfloor (\log _2 x) / 2 \rfloor$. Figure 6 shows a plot of the deviations of these seeds from the actual square root value. As depicted in the figure, the use of $s_0$ provides the most accurate initial estimation of the actual square root value. The impact of using the aforementioned seeds on the accuracy results is presented in Table 6. The results show that $s_0$ yields the best accuracy results.

**Table 6:** The impact of using different seeds on the accuracy results of our proposed algorithm with a LUT size of 1 KB.

Square root method	Seed	MRE	Accuracy (bits)
Dianov et al.	$3 \times 2^{k-1}$	$4.8894 \times 10^{-4}$	11.00
Blinn	Eq. (11)	$2.7264 \times 10^{-4}$	11.84
Proposed algorithm	$s_0$	$2.1683 \times 10^{-4}$	12.17

The proposed algorithm utilizing a 1-KB lookup table is also compared to six of the reviewed algorithms in terms of MRE and accuracy values. The obtained results are provided in Table 7. The nonrestoring method is selected as a baseline for this comparison with the best MRE of $8 \times 10^{-8}$. However, this high accuracy is obtained with the worst performance in terms of number of clock cycles, as displayed in Table 4. Our proposed square rooting algorithm comes in second place with a MRE of $2.17 \times 10^{-4}$ and 12.17 bits of accuracy. The latter value is achieved with fewer numbers of clock cycles when compared to Newton–Raphson’s and Bakhshali’s methods. As observed from this table, our proposed algorithm shows a balanced tradeoff between accuracy and number of clock cycles among the selected square rooting methods. We also note that providing accuracy values between 10 and 15 bits may be sufficient for some applications that are related to the Internet of Things (IoT) and machine learning. These applications are known as error resilient because the reduced accuracy does not negatively impact the generated results^26,63^. Furthermore, a recent trend in machine learning, exemplified by the use of specialized data formats such as the deep-learning float (DLFloat) and the Brain float (bfloat16), confirms the practical value in using reduced accuracies in certain application contexts. An accrued benefit of this trend is the realization of significant savings in power and energy requirements^64,65^.

**Table 7:** Maximum relative error and accuracy values of selected methods including our proposed algorithm for square rooting. The number of clock cycles, required by each method, is also provided for comparison purposes. For our proposed algorithm, a LUT size of 1 KB and a 16-bit precision of $\cos \theta$ values are assumed.

Square root method	Clock cycles	MRE	Accuracy (bits)
Nonrestoring	313	$8.00 \times 10^{-8}$	23.58
Newton–Raphson	89	$3.20 \times 10^{-3}$	8.29
Bakhshali	92	$3.20 \times 10^{-3}$	8.29
Goldschmidt	35	$9.70 \times 10^{-3}$	5.69
Polynomial approximation	39	$2.90 \times 10^{-2}$	5.11
Dianov et al.	34	$5.00 \times 10^{-3}$	7.64
Proposed algorithm	85	$2.17 \times 10^{-4}$	12.17

### Impact of seed selection on the number of iterations

In this section, we analyze the impact of using our seed generation approach on the number of iterations for two iterative methods and compare our results against other selected seed values. In order to summarize these results using one metric, we computed the weighted average number of iterations as follows:

19 $$\begin{aligned} I_{avg} = \sum \limits _{i=1}^{I_{max}} w_i \times i, \end{aligned}$$

where $I_{max}$ is equal to the maximum number of iterations needed to calculate all the square root values of *x* and $w_i$ is equal to the fraction of calculated square roots in iteration *i*.

#### Newton–Raphson’s method

Although the equation for the Newton–Raphson’s method given in Eq. (6) does not include a division operation when compared with the original method in Eq. (5), the latter is selected in this analysis due to the fact that Eq. (6) requires an inverse square root as a seed.

**Figure 7:**
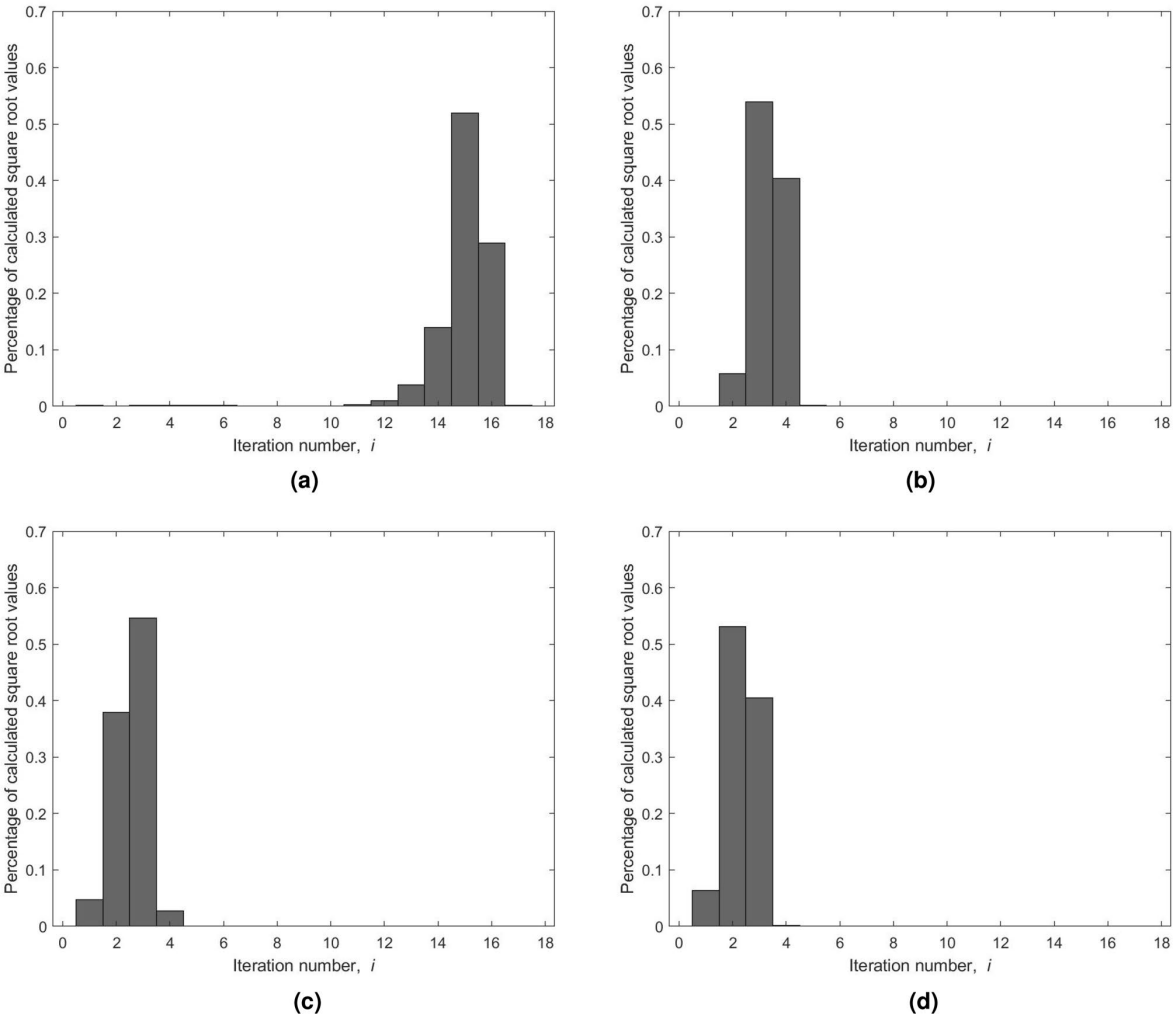
Distribution of the number of iterations of Newton–Raphson’s method using different seed values: (**a**) seed is equal to *x*. (**b**) Seed is equal to $3 \times 2^{k-1}$. (**c**) Seed using Blinn’s method. (**d**) Seed is obtained using our algorithm based on bit manipulation, $s_0$.

For any positive integer *x* up to $2^{24}-1$ and assuming a four-decimal-digit accuracy for the square root value, we analyzed the impact of using different seed values on the number of iterations for the Newton–Raphson’s method. Four variations are considered: (a) the seed is initialized with *x* as a baseline for this comparison; (b) the seed reported by Dianov et al.^52^; (c) the seed is initialized using Blinn’s method; and (d) the seed is obtained using our bit-manipulation approach, $s_0$. The obtained results are reported in Fig. 7. As illustrated in Fig. 7a, the majority of square root values are obtained in 15 iterations when the seed value is set to *x*. Besides, $I_{max}$ is equal to 17 for this specific range and assumed accuracy. A significant reduction of 77.74% is realized using the seed $3 \times 2^{k-1}$ with a maximum number of iterations equals to five. This outcome is shown in Fig. 7b. When using a seed from Blinn’s method, the required average number of iterations is further reduced with $I_{max}$ being equal to four (see Fig. 7c). Overall, a reduction of nearly 83% in the average number of iterations, $I_{avg}$, is realized compared to the baseline seed *x*. Our approach for seed generation yields further improvements of 30% and 8.33% in $I_{avg}$ to the results obtained by employing variations (b) and (c), respectively. As disclosed in Fig. 7d, the majority of the square root values are now generated after only two iterations with $I_{max}$ being the same as when using Blinn’s seed. Overall, our approach for seed selection led to a major reduction in the number of iterations required by Newton–Raphson’s method. In particular, our approach achieved the lowest $I_{avg}$ of 2.3409 iterations. In Table 8, we present both the $I_{avg}$ and $I_{max}$ values for the four considered variations.

**Table 8:** Impact of different seed selections on the number of iterations for Newton–Raphson’s method using an accuracy of four decimal digits.

Seed variation	Seed value	${I}_{{avg}}$	${I}_{{max}}$	Reduction in ${I}_{{avg}}$
(a)	*x*	15.0288	17	NA
(b)	$3 \times 2^{k-1}$	3.3448	5	77.74%
(c)	Blinn’s method	2.5535	4	83.33%
(d)	Proposed seed $(s_0)$	2.3409	4	84.42%
				(30.01% from Dianov et al. and 8.33% from Blinn)

#### Bakhshali’s method

**Figure 8:**
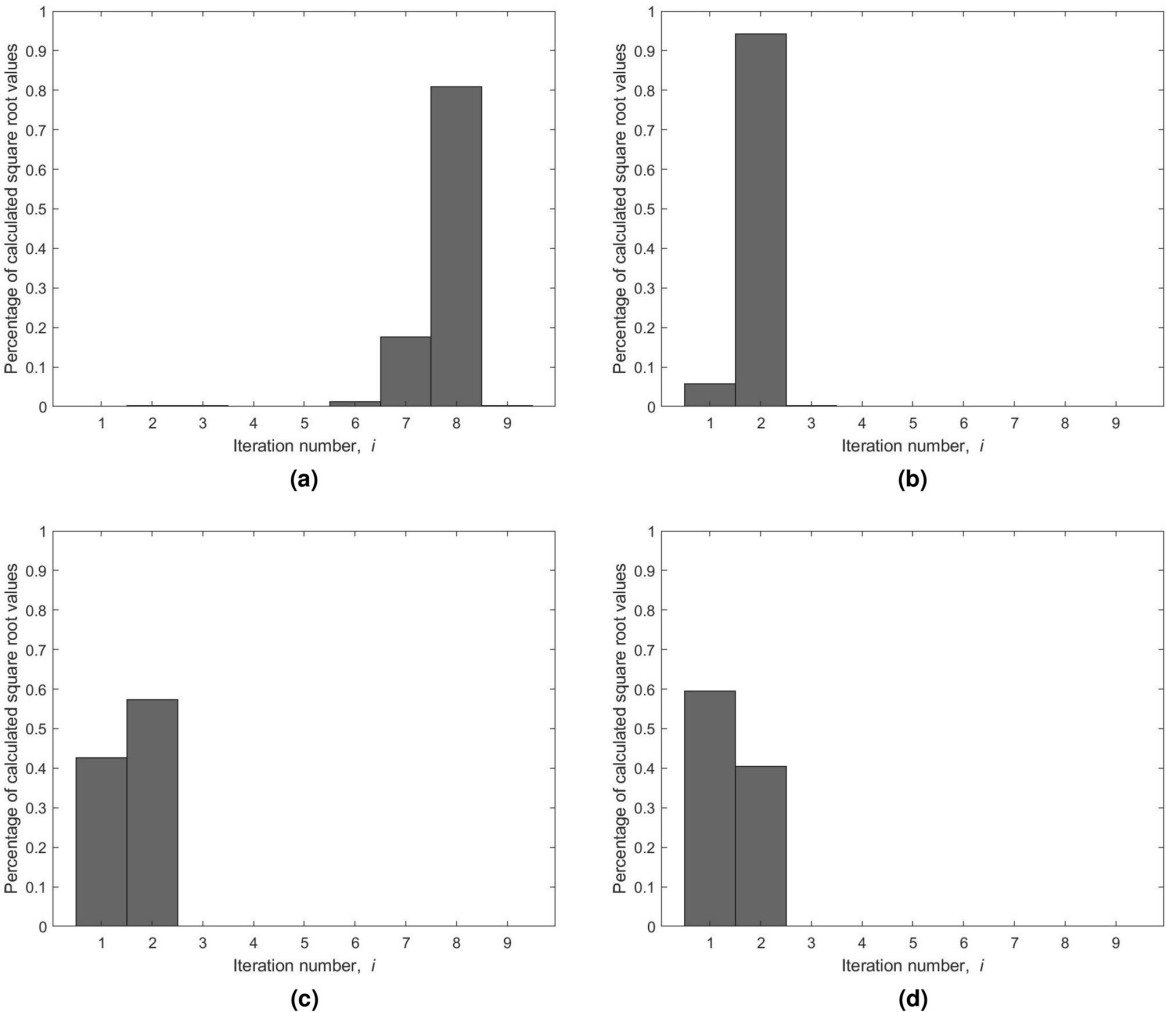
Distribution of the number of iterations of Bakhshali’s method using different seed values: (**a**) Seed is equal to *x*. (**b**) Seed is equal to $3 \times 2^{k-1}$. (**c**) Seed using Blinn’s method. (**d**) Seed is obtained using our algorithm based on bit manipulation, $s_0$.

The impact of using the same four variations of seed values on the number of iterations is also examined for Bakhshali’s method (see Fig. 8 for results). When the seed is equal to *x*, Bakhshali’s method calculates most of the square root values in the eighth iteration. The maximum number of iterations $I_{max}$ is reduced from 17 to 9 when compared to Newton–Raphson’s method. As expected, the maximum number of iterations is reduced from 9 to 3 using $3 \times 2^{k-1}$ as a seed. Blinn’s method presents further improvement to the maximum number of iterations by reducing it to two. Likewise, our bit-manipulation approach produces the entire set of square root values in just two iterations. The majority of the square root values are calculated after the first iteration in contrast to Blinn’s, where the most values are computed after the second iteration. As a result, the weighted average number of iterations $I_{avg}$ is equal to 1.5737 when using Blinn’s method and 1.4052 using our proposed approach for seed calculation, $s_0$. This translates to a reduction of 10.71% when compared to the seed provided by Blinn’s method. By employing our seed generation mentioned as variation (d), the average number of iterations reaches its smallest value of 1.4052 and is reduced by 81.97%. Overall, our approach for seed selection led to a major reduction in the number of iterations required by Bakhshali’s method in terms of both $I_{avg}$ and $I_{max}$ values. In Table 9, we present these values for the four considered variations.

**Table 9:** Impact of different seed selections on the number of iterations for Bakhshali’s method using an accuracy of four decimal digits.

Seed variation	Seed value	${I}_{{avg}}$	${I}_{{max}}$	Reduction in ${I}_{{avg}}$
(a)	*x*	7.7945	9	NA
(b)	$3 \times 2^{k-1}$	1.9421	3	75.08%
(c)	Blinn’s method	1.5737	2	79.81%
(d)	Proposed seed $(s_0)$	1.4052	2	81.97%
				(27.65% from Dianov et al. and 10.71% from Blinn)

## Conclusion

In this paper, a novel seed-generation technique followed by a novel quadrature-based square rooting algorithm are proposed. The seed generation approach is based on bit manipulation and requires only one addition operation and a single right shift. The proposed seed yields a larger reduction in the number of iterations for Newton–Raphson’s and Bakhshali’s methods than when compared to the seed reported by Dianov et al. as well as that obtained by Blinn’s method. Additionally, we describe a quadrature-based algorithm for computing the square root of unsigned numbers yielding a maximum relative error of $2.17 \times 10^{-4}$ and requiring only a single iteration. The obtained accuracy of this approximation shows a significant improvement compared to Newton–Raphson’s and Bakhshali’s methods. In addition, it yields a reduced latency measured as the total number of clock cycles. As part of our future work, we plan to evaluate the performance of our square root approximation algorithm in terms of computational time. To properly undertake this part of our research, we aim to implement all the listed methods in both software and hardware; the latter by employing hardware accelerators such as FPGAs or GPUs^66^. This is because some of these techniques are more suitable for hardware than software and vice versa. Further, we anticipate employing the proposed square rooting algorithm in signal processing applications of remotely sensed hyperspectral images^67^.

## Supplementary Information

Supplementary Information.

## Data Availability

All data generated or analyzed during this study are included in this published article.
