# Supplementary material for: Novel seed generation and quadrature-based square rooting algorithms
Source: Sci Rep. 2022 Nov 29;12:20540. doi: 10.1038/s41598-022-25039-y (PMC9708849; doi:10.1038/s41598-022-25039-y)
Supplement: Supplementary file 1 — Supplementary Information. [file 41598_2022_25039_MOESM1_ESM.pdf]

Appendix A

We provide in Table S1 below the first three occurrences of deviations in the values of the initially estimated seed, s_0, from the square root of x, with x being a square unsigned integer number. The value of a deviation is computed as $|s_0 - \sqrt{x}|$.

Square number, x	Exact square root ($\sqrt{x}$)	Initial estimation of seed value (s_0)	Deviation of s_0 from $\sqrt{x}$
1	1	1	0
4	2	2	0
9	3	3	0
...	...	...	...
400	20	20	0
441	21	21	0
484	22	23	1
529	23	24	1
576	24	25	1
625	25	25	0
676	26	26	0
729	27	27	0
...	...	...	...
1444	38	38	0
1521	39	39	0
1600	40	41	1
1681	41	42	1
1764	42	43	1
1849	43	44	1
1936	44	46	2
2025	45	47	2
2116	46	48	2
2209	47	49	2
2304	48	50	2
2401	49	50	1
2500	50	51	1
2601	51	52	1
2704	52	53	1
2809	53	53	0
2916	54	54	0
3025	55	55	0
...	...	...	...
5476	74	74	0
5625	75	75	0
5776	76	77	1
5929	77	78	1
6084	78	79	1
6241	79	80	1
6400	80	82	2
6561	81	83	2
6724	82	84	2
6889	83	85	2
7056	84	87	3
7225	85	88	3
7396	86	89	3
7569	87	91	4
7744	88	92	4
7921	89	93	4
8100	90	95	5
8281	91	96	5
8464	92	97	5
8649	93	97	4
8836	94	98	4
9025	95	99	4
9216	96	100	4
9409	97	100	3
9604	98	101	3
9801	99	102	3
10000	100	103	3
10201	101	103	2
10404	102	104	2
10609	103	105	2
10816	104	106	2
11025	105	107	2
11236	106	107	1
11449	107	108	1
11664	108	109	1
11881	109	110	1
12100	110	111	1
12321	111	112	1
12544	112	113	1
12769	113	113	0
12996	114	114	0
13225	115	115	0
...	....	...	...

Table S1. First three occurrences of deviations of the initially estimated seed values (s_0) from the exact square root of a square unsigned number x.
